# Propofol protects cardiomyocytes from doxorubicin-induced toxic injury by activating the nuclear factor erythroid 2-related factor 2/glutathione peroxidase 4 signaling pathways

**DOI:** 10.1080/21655979.2022.2036895

**Published:** 2022-04-01

**Authors:** Ziyun Lu, Zhiyi Liu, Bei Fang

**Affiliations:** Department of Anesthesiology, The First Affiliated Hospital of Nanchang University, Nanchang, China

**Keywords:** Propofol, Nrf2/GPx4, ferroptosis, inflammation, cardiomyocyte

## Abstract

Propofol offers important protective effects in ischemia/reperfusion-induced cardiomyocyte injury, but its specific mechanisms in doxorubicin (DOX)-induced cardiotoxicity have not been investigated. In this paper, we attempted to explore the effects of propofol on DOX-induced human cardiomyocyte injury and its related mechanisms. H9c2 cell viability was assessed by cell counting kit-8 and lactate dehydrogenase assay kit. Nuclear factor erythroid 2-related factor 2 (NRF2)/glutathione peroxidase 4 (GPx4) signaling pathway-related protein levels were measured by Western blot. Ferroptosis was evaluated by corresponding kits and Western blot and apoptosis was detected by CCK-8, terminal deoxynucleotidyl transferase dUTP nick-end labeling and Western blot. Oxidative stress was assessed by reactive oxygen species kit and the commercial kits, and inflammation response was analyzed by enzyme-linked immunosorbent assay and Western blot. The results showed that propofol attenuated DOX-induced cytotoxicity and activated Nrf2/GPx4 signaling pathways in H9c2 cells. In addition, propofol also alleviated DOX-induced ferroptosis, increased cell viability and inhibited apoptosis, oxidative stress and inflammatory responses in H9c2 cells through activation of Nrf2/GPx4 signaling pathways. In summary, propofol provides the protection against DOX-induced cardiomyocyte injury by activating Nrf2/GPx4 signaling, providing a new approach and theoretical basis for the repair of cardiomyocytes.

## Graphical abstract

**Figure:**
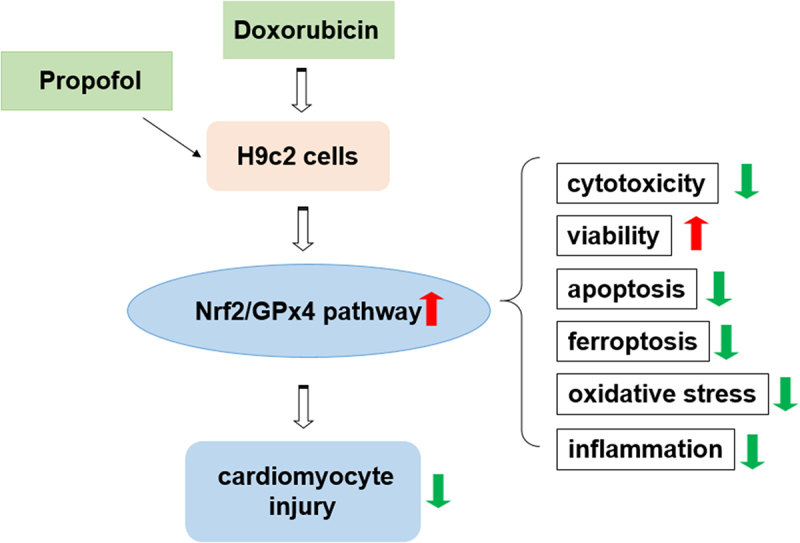

## Introduction

Cardiomyocyte injury is the main cause of heart dysfunction and even heart failure [1,2]. During the treatment of cancer disease, chemotherapy based on anthracyclines such as doxorubicin (DOX) can lead to the occurrence of cumulative and progressive cardiomyopathy [3]. This is because DOX can trigger mitochondrial damage, leading to apoptosis and excessive production of ROS [4]. Oxidative stress also directly promotes the expression of inflammatory cytokines, thereby amplifying the inflammatory process [5]. In addition, DOX can interact with iron to produce hydroxyl radicals, which can cause cellular damage [6], suggesting that DOX-induced cardiotoxicity can lead to another form of cell death-ferroptosis [7]. Accordingly, cardiomyocyte apoptosis, oxidative stress, inflammation and subsequent ferroptosis have been associated with DOX-induced cardiomyocyte injury [8,9]. Also, preventing cardiomyocyte damage and even death has been recognized as an effective cardioprotective strategy [10].

Propofol (2,6-diisopropylphenol) is one of the most frequent intravenous anesthetic agents in modern medicine [11]. Propofol’s phenolic structure allows it to provide potent anesthetic sedation while preventing I/R-induced cardiomyocyte injury. For example, propofol protects H9c2 cells from oxygen glucose deprivation and I/R injury [12]. Propofol achieves protection against I/R-induced cardiomyocyte injury by reducing apoptosis and NF-κB nuclear translocation in cardiomyocytes through the ERK signaling pathway [13]. Propofol supplementation in ECG has heart protective effect on I/R injury [14]. Several investigations looked at the effect of propofol on cardio-protection in ANT-induced cardiotoxicity [15]. However, a systematic understanding of how propofol provides protection against DOX-induced cardiomyocyte injury is still lacking. It has previously been observed that propofol could activate Nrf2 signaling in hydrogen peroxide-induced H9c2 cells [16]. And the enhanced Nrf2 signaling could inhibit ferroptosis in DOX-induced cardiomyocytes [17]. Consequently, this paper speculated that propofol may mitigate DOX-induced cardiomyocyte injury by activating Nrf2/GPx4 signaling to attenuate ferroptosis, providing a meaningful therapeutic means to protect cardiomyocytes from DOX-induced cytotoxicity.

In this study, we aimed to identify the effects of propofol on DOX-induced human cardiomyocyte injury and its related mechanisms. We hypothesized that propofol may play a protective role in DOX-induced cardiomyocyte injury by activating Nrf2/GPx4 signaling. We first established an *in vitro* model of DOX-induced cardiomyocyte injury. Subsequently, we observed the cytotoxic changes of H9c2 cells after DOX and propofol treatment. Subsequently, cell apoptosis, oxidative stress, inflammation and ferroptosis were explored in H9c2 cells under DOX and DOX+propofol conditions after the addition of Nrf2 inhibitor ML385, respectively. These detailed experimental data provided some new insights into the recovery of cardiomyocyte injury.

## Materials and methods

### Cell culture and treatment

Rat cardiomyocyte cell line H9c2 cells (Procell, Wuhan, China) were placed in Dulbecco’s modified Eagle’s Medium (DMEM; Gibco) with a final concentration of 10% fetal bovine serum (FBS; Hyclone, South Logan, USA) and 1% penicillin/streptomycin (both Gibco) for culturing. The incubator temperature was set at 37°C and supplied with 5% CO_2_ and 95% air.

Part of the H9c2 cells were treated with only 1 μM of DOX (Sigma-Aldrich) for 24 h for the establishment of an *in vitro* cardiomyocyte injury model [18]. While another part of H9c2 cells were first pretreated with 5, 10 and 20 μM propofol (Sigma-Aldrich) for 1 h and then incubated with 1 μM DOX for 24 h. The control group was left untreated. H9c2 cells treated in each group were harvested for later experiments.

### Cell counting kit (CCK)-8 assay

The assessment of cell viability in H9c2 cells was carried out with the adoption of CCK-8 method [19]. H9c2 cells treated accordingly in each group were inoculated in 96-well plates at 37°C in 5% CO_2_ for 24 h. Next, each well was added with 10 μL of CCK-8 solution (GlpBio, Montclair, CA, USA) using a repetitive pipette and incubated for 2 h, strictly comply with the manufacturer’s notes. Determination of absorbance in each well at 450 nm was performed utilizing an enzyme marker (Molecular Devices, Shanghai, China).

### Lactate dehydrogenase (LDH) activity assay

The release of LDH activity was determined by means of LDH assay kit (Solarbio, Beijing, China) for assessing the cell injury in H9c2 cells [20]. Cells were centrifuged at 8000 g for 10 min at 4°C and the supernatant was collected on ice for testing. 200 μL of LDH reagent solution was added in line with the instructions provided by vendor. Finally, the measurement of absorbance was performed at 450 nm with a spectrophotometer (Mapada, Shanghai, China) which had been preheated for 30 min.

### Western blot assay

Following the treatment with different stimulus, H9c2 cells were lysed with a frozen radioimmunoprecipitation (RIPA; Beyotime, Shanghai, China) buffer containing protease inhibitors phenylmethylsulfonyl fluoride (PMSF). Then, the protein concentration was determined by bicinchoninic acid (BCA) protein assay kit (Beyotime, Shanghai, China). Each protein sample (30 μg) was separated using 15% sodium dodecyl sulfate-polyacrylamide gel electrophoresis (SDS-PAGE). The polyvinylidene difluoride (PVDF) membrane which was loaded with the protein samples were first blocked with 5% nonfat milk and then incubated overnight at 4°C with the primary antibodies against Nrf2 (nucleus), Nrf2 (cytoplasm), Keap1, GPx4, PTGS2, Ferritin, ASCL4, Bax, Bad, Bcl-2, p-p65, followed by the incubation with secondary antibodies for another 1 h. The protein bands were identified with the application of Odyssey Infrared Imaging System (LI-COR Biosciences, Lincoln, NE, USA).

### Detection of Fe^2+^ content

Based on the protocol summary of Fe^2+^ assay, the samples were placed into 96-well plates. Each well was added with 5 µL assay buffer (Abcam, Cambridge, MA, USA) and incubated for 30 min at 37°C. Then, 100 µL Iron Probe (BioAssay Systems, CA, USA) was added to each well containing the Iron Standard and test samples and incubated for 1 h at 37°C protected from light. The absorbance at 593 nm was analyzed immediately with a colorimetric microplate reader (Bio-Rad Laboratories, Shanghai, China).

### Terminal deoxynucleotidyl transferase dUTP nick-end labeling (TUNEL) assay

For the evaluation of cell apoptosis, TUNEL staining was conducted in line with the procedures of manuals [21]. After fixing with PBS containing 1% paraformaldehyde, TUNEL staining of H9c2 cells was performed using the ApopTag® Plus Fluorescein In Situ Apoptosis Detection Kit (Millipore, USA). The nuclei of H9c2 cells were stained with DAPI. After that, the stained cells were observed and captured employing an Olympus DX51 fluorescence microscope (Olympus, Tokyo, Japan).

### Reactive oxygen species (ROS) level assessment

ROS level in H9c2 cells were examined by 2’,7’-dichlorodihydrofluorescein diacetate (DCFH-DA; MedChemExpress, Shanghai, China). H9c2 cells treated with different stimulus were incubated with 25 μM DCFH-DA at 37°C in the dark. Then, the cells were washed with PBS for three times before the fluorescence was observed with a microplate reader (Bio-Rad).

### Malondialdehyde (MDA), glutathione (GSH) and 4-HNE levels detection

The content of MDA was measured using the Lipid Peroxidation MDA Assay Kit (Beyotime). The level of GSH was examined by the use of the Micro Reduced GSH Assay Kit (Solarbio, Beijing, China). The 4-HNE level was detected with the Lipid Peroxidation 4-HNE Assay kit (Abcam). Application of all reagents was performed in strict accordance with standard operating procedures.

### Enzyme-linked immunosorbent assay (ELISA)

The levels of inflammatory cytokines TNF-α, IL-6 and IL-1β in H9c2 cells were assessed by the Rat TNF-α/IL-6/IL-1β ELISA Kit (Gefan biotechnology, Shanghai, China) following the standard procedures recommended by the manufacturer [22]. Briefly, the samples were incubated with 100 µl/well biotinylated antibody for 1 h, then labeled with 100 µl/well Streptavidin for 20 min. Finally, the incubation of samples and TMB solution was carried out for another 20 min prior to measuring A450 with a microplate reader (Bio-Rad). Experiment was carried out three times.

### Statistical analysis

All data were documented by the way of the mean ± standard deviation (SD). Statistical analyses were conducted with the application of SPSS 19.0 (SPSS Inc., Chicago, IL, USA). The data were in accordance with the normal distribution by Shapiro-Wilk test. The comparisons were presented using one-way ANOVA with a post hoc Bonferroni multiple comparison test. The differences were considered to be statistically significant when P was less than 0.05.

## Results

In this study, we explored the biological effects of propofol and the potential mechanism in DOX-induced H9c2 cells. The data showed that the propofol inhibited DOX-induced cytotoxicity and activates Nrf2/GPx4 signaling pathway. In addition, propofol attenuates ferroptosis and apoptosis, but promotes cell viability in DOX-induced H9c2 cells. Moreover, Propofol alleviates DOX-induced oxidative stress and inflammatory response in H9c2 cells.

### Propofol alleviates DOX-induced cytotoxicity and activates Nrf2/GPx4 signaling pathway in H9c2 cells

**Figure 1.**
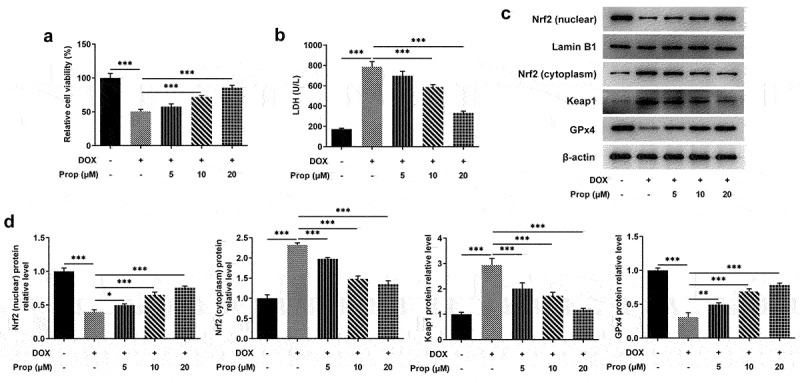
Propofol alleviates DOX-induced cytotoxicity and activates Nrf2/GPx4 signaling pathway in H9c2 cells. (a) Relative cell viability of H9c2 cells in the groups of control, DOX, DOX+Prop (5, 10, 20 μM) was examined at 24 h by CCK-8. ***P < 0.001. (b) The level of LDH in the groups of control, DOX, DOX+Prop (5, 10, 20 μM) was detected by LDH assay kit. ***P < 0.001. (c-d) The protein levels of Nrf2 (nuclear), Nrf2 (cytoplasm), Keap1 and GPx4 in H9c2 cells were measured by Western blot in the groups of control, DOX, DOX+Prop (5, 10, 20 μM). *P < 0.05, **P < 0.01, ***P < 0.001.

To assess the impacts of propofol on DOX-induced viability and Nrf2/GPx4 signaling pathway in H9c2 cells, we first determined the viability of H9c2 cells in each group. Figure 1(a) shows that the viability of H9c2 cells decreased by approximately 50% in DOX-induced H9c2 cells compared with the control group but rose gradually with the increasing of propofol concentration. Figure 1(b) illustrates that LDH level increased dramatically in DOX-induced H9c2 cells compared with the control group but decreased steadily in a concentration-dependent manner in the groups of DOX+Prop (5, 10 and 20 μM). Next, Western blot assay identified the influence of propofol on Nrf2/GPx4 signaling pathway. As shown in Figure 1(c,d), the protein levels of Nrf2 (nucleus) and GPx4 related to Nrf2/GPx4 signaling were declined rapidly after DOX treatment but rose again in DOX+Prop groups, by contrast to the control group. While the protein levels of Nrf2 (cytoplasm) and Keap1 presented a completely opposite trend to those of Nrf2 (nucleus) and GPx4. Since propofol showed better efficiency at a concentration of 20 μM, the DOX+Prop (20 μM) group was chosen for the next experiment. In general, therefore, it seems that propofol not only protects H9c2 cells largely from DOX-induced cytotoxicity, but also activates the Nrf2/GPx4 signaling pathway.

### Propofol attenuates DOX-induced ferroptosis in H9c2 cells by activating Nrf2/GPx4 signaling pathway

**Figure 2.**
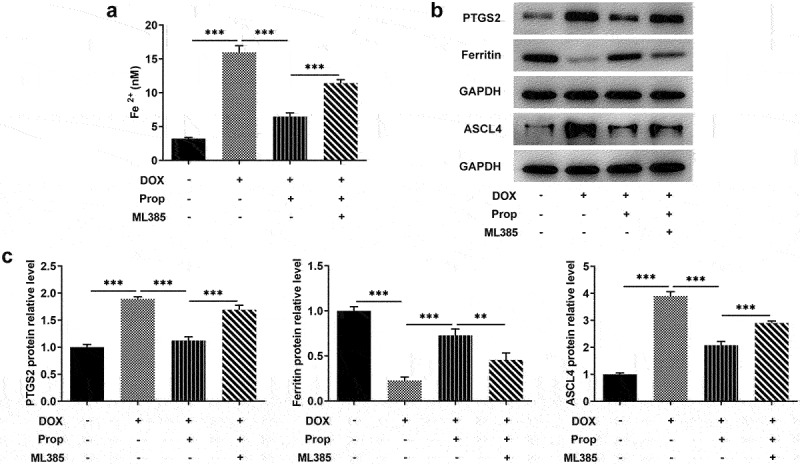
Propofol attenuates DOX-induced ferroptosis in H9c2 cells by activating Nrf2/GPx4 signaling pathway. (a) The content of Fe^2+^ in H9c2 cells was assessed by the commercial kit in the groups of control, DOX, DOX+Prop (5, 10, 20 μM). ***P < 0.001. (b-c) The protein levels of PTGS2, Ferritin and ASCL4 related to ferroptosis in H9c2 cells were detected by Western blot in the groups of control, DOX, DOX+Prop (5, 10, 20 μM). **P < 0.01, ***P < 0.001.

Propofol was confirmed to activate Nrf2/GPx4 signaling in the last set of experiments. Here, the experiments in this section sought to further discover the action of propofol on ferroptosis in DOX-induced H9c2 cells. Figure 2(a) presents a steep rise of the content of Fe^2+^ in the DOX group (vs Control) and a sharp drop in the DOX+Prop group, while the Nrf2 signaling inhibitor ML385 reversed the result partly. Further assays in Figure 2(b,c) revealed that after DOX treatment in H9c2 cells, the protein levels of PTGS2 and ASCL4 related to ferroptosis were elevated markedly (vs Control), and decreased obviously in the DOX+Prop group, but rose again after addition of ML385. In addition, the trend of Ferritin level in each group was opposite to the changes in PTGS2 and ASCL4. The results indicate that propofol could reduce DOX-induced ferroptosis in H9C2 cells through the activation of Nrf2/GPx4 signaling.

### Propofol promotes DOX-induced viability and inhibits apoptosis in H9c2 cells through the activation of Nrf2/GPx4 signaling pathways

**Figure 3.**
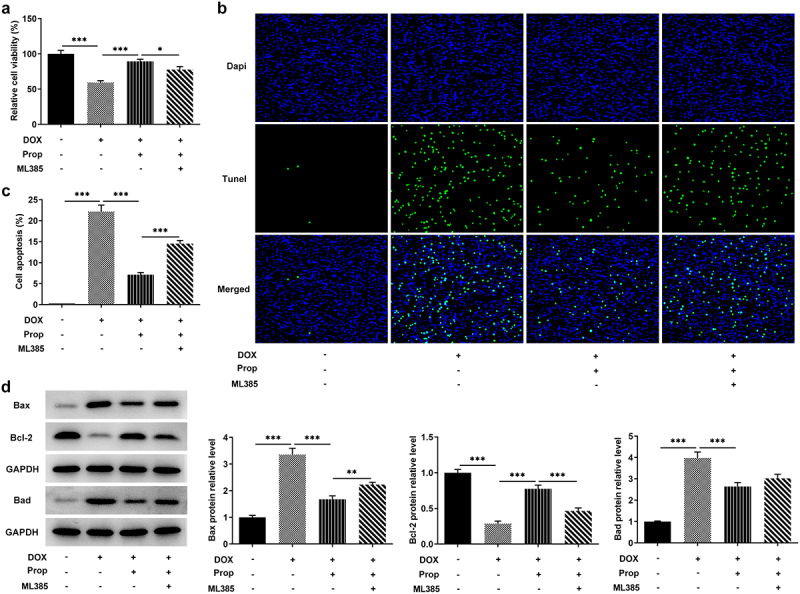
Propofol promotes DOX-induced viability and inhibits apoptosis in H9c2 cells through the activation of Nrf2/GPx4 signaling pathway. (a) Relative cell viability of H9c2 cells was measured by CCK-8 in the groups of control, DOX, DOX+Prop and DOX+Prop+ML385. *P < 0.05, ***P < 0.001. (b-c) Cell apoptosis rate of H9c2 cells was examined by TUNEL in the groups of control, DOX, DOX+Prop and DOX+Prop+ML385. ***P < 0.001. (d) Protein levels of Bax, Bad and Bcl-2 related to apoptosis were tested by Western blot of H9c2 cells was measured by CCK-8 in the groups of control, DOX, DOX+Prop and DOX+Prop+ML385. **P < 0.01, ***P < 0.001.

This set of experiments explored the impact of propofol on viability and apoptosis in DOX-induced H9c2 cells. In Figure 3(a), we can see that DOX induced a lower cell viability compared to the control group, but the addition of propofol led to a successful rise in viability, but ML385 kept cell viability at a lower level again. Likewise, as shown in Figure 3(b,c), more TUNEL-positive H9c2 cells in the DOX group (vs Control), obviously less TUNEL-positive cells in the DOX+Prop group, as well as slightly increased TUNEL-positive cells in the DOX+Prop +ML385 group were observed. Accordingly, DOX induced a high apoptosis rate (vs Control), while propofol resulted in a significant reduction in apoptosis rate, but ML385 increased the apoptosis rate. Western blot in Figure 3(d) also detected rapidly elevated protein levels of pro-apoptotic Bax and Bad and the declined protein level of anti-apoptotic markers Bcl-2 in the DOX group (vs Control), as well as an increase in the expression of Bax and Bad and a reduced expression of Bcl-2 in the DOX+Prop group, whereas ML385 increased the levels of Bax and Bad and decreased the levels of Bcl-2. These findings suggest that, in general, propofol in H9c2 cells could remarkably prevent DOX-induced loss of cell viability and inhibit cardiomyocyte apoptosis via activating of Nrf2/GPx4 signaling pathways.

### Propofol inhibits DOX-induced oxidative stress in H9c2 cells via the activation of Nrf2/GPx4 signaling pathways

**Figure 4.**
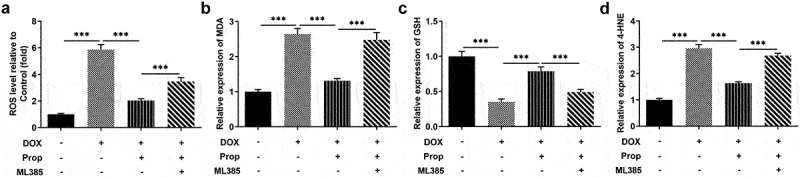
Propofol inhibits DOX-induced oxidative stress in H9c2 cells via the activation of Nrf2/GPx4 signaling pathway. (a) ROS level of H9c2 cells was determined by DCFH-DA kit in the groups of control, DOX, DOX+Prop and DOX+Prop+ML385. ***P < 0.001. (b-d) The levels of MDA, GSH and 4-HNE were measured by the corresponding commercial kits in the groups of control, DOX, DOX+Prop and DOX+Prop+ML385. ***P < 0.001.

The impact of propofol on DOX-induced oxidative stress in H9c2 cells was tested using the corresponding kits. It can be seen from the data in Figure 4(a) that the stimulation of DOX largely increased the content of oxidative stress marker ROS in H9c2 cells (vs Control), which was brought down by propofol. While there was a slight rise in the level of ROS after addition of ML385. Further examination was carried out and closer inspection of Figure 4(b-d) discovered that the levels of MDA and 4-HNE were clearly enhanced by DOX induction in H9c2 cells, but decreased by propofol, suggesting that propofol attenuated DOX-induced oxidative stress. However, the addition of ML385 made those of MDA and 4-HNE elevated again. More importantly, the level of GSH presented an opposite trend to those of MDA and 4HNE. An implication of this is the fact that propofol suppresses DOX-induced oxidative stress in H9c2 cells through the activation of Nrf2/GPx4 signaling pathways.

### Propofol inhibits DOX-induced inflammatory response in H9c2 cells via activating Nrf2/GPx4 signaling pathways

**Figure 5.**
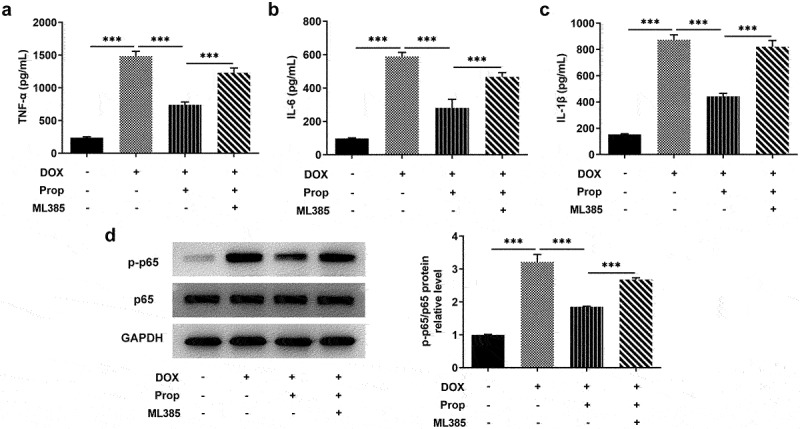
Propofol inhibits DOX-induced inflammatory response in H9c2 cells via activating Nrf2/GPx4 signaling pathway. (a-c) The levels of TNF-α, IL-6, IL-1β were examined by ELISA assay in the groups of control, DOX, DOX+Prop and DOX+Prop+ML385. ***P < 0.001. (d) Protein levels of p-p65 and p65 were assessed by Western blot in the groups of control, DOX, DOX+Prop and DOX+Prop+ML385. ***P < 0.001.

The determination of inflammation in DOX-induced H9c2 cells employed the assays of ELISA and Western blot. It is apparent from Figure 5(a-c) that there was a clear trend of increasing in the levels of inflammatory cytokines TNF-α, IL-6 and IL-1β after DOX induction in H9c2 cells, whereas propofol decreased the levels of TNF-α, IL-6 and IL-1β. ML385 offset the inhibitory effect of propofol on DOX-induced H9c2 cells. As can be seen from Figure 5(d), the phosphorylation level of P65 was significantly elevated by DOX stimulation in H9c2 cells, but propofol decreased its level substantially, yet ML385 made its level elevated again. Overall, these findings strengthen the idea that propofol could inhibit DOX-induced inflammation in H9c2 cells via activating Nrf2/GPx4 signaling pathways.

## Discussion

Cardiotoxicity induced by DOX in anticancer therapy can lead to myocardial cell damage and subsequent left ventricular dysfunction, dilated cardiomyopathy and heart failure [23]. Previous studies have shown that ferroptosis, apoptosis, oxidative stress and inflammation are key components of DOX-induced cardiomyocyte injury [8,9]. Propofol serves a pivotal role in I/R-induced cardiomyocyte injury [24]. As a result, with a view to exploring the role of propofol in DOX-induced cardiomyocytes, the article made efforts in terms of ferroptosis, apoptosis, oxidative stress and inflammation and found that propofol reduced DOX-induced H9c2 cytotoxicity and activated Nrf2/GPx4 signaling. In addition, propofol attenuated DOX-induced ferroptosis in H9c2 cells by activating Nrf2/GPx4 signaling, increased cell viability, and inhibited the apoptosis, oxidative stress, and inflammatory responses.

Propofol is an intravenous agent often applied to maintain sedation in children for anesthesia, surgery and intensive care [25]. Propofol has been reported to attenuate the cytotoxicity of rat astrocytes induced by tert-butyl hydroperoxide [26], suggesting the inhibition of propofol on cytotoxicity. Our study utilized 1 μM DOX to induce H9c2 cells for 24 h to establish an *in vitro* cardiomyocyte injury model. Subsequent assay for cytotoxicity revealed that DOX induced more severe cytotoxicity. But later addition of propofol at concentrations of 5, 10, and 20 μM revealed a reduction in the cytotoxicity of H9c2 cells, implying that propofol was effective in reducing DOX-induced cytotoxicity. Moreover, recent evidence suggests that propofol promotes the proliferation and invasion of gallbladder cancer cells through activation of Nrf2 signaling [27]. Moreover, mitochondria-dependent ferroptosis plays a key role in DOX-induced cardiotoxicity, of which GPx4 is a key regulator [9]. In this study, elevated levels of Nrf2/GPx4 signaling after propofol treatment in DOX-induced H9c2 cells also indicate that propofol could activate Nrf2/GPx4 signaling.

Ferroptosis is a new form of cell death characterized by iron-dependent accumulation of lipid peroxides to lethal levels [28]. Some studies have shown that increased iron in the body contributes to DOX-induced cardiotoxicity [29]. This is due to the fact that in DOX-induced cardiac injury, the expression of inducible heme oxygenase Hmox1 is increased by Nrf2, which catalyzes heme degradation and promotes the release of free iron, giving rise to ferroptosis and ultimately heart failure [7]. Our experiments examined the levels of Fe ions and the expression of ferroptosis-associated proteins in DOX-induced H9c2 cells. An increase in Fe^2+^ and an increase in the levels of ferroptosis-associated PTGS2 and ASCL4 were found, whereas the serum marker of total body iron stores Ferritin was decreased, indicating that DOX induced ferroptosis in H9c2 cells. Furthermore, propofol has been demonstrated to modulate Nrf2/GPx4 signaling in this study, and Nrf2 regulates the content of GPX4 protein and intracellular free Fe^2+^ directly or indirectly [^30–33^]. Therefore, the article assessed again the effect of propofol on ferroptosis. Reduced levels of Fe^2+^ and ferroptosis proteins PTGS2 and ASCL4, as well as increased level of ferritin were observed, indicating that propofol inhibited ferroptosis in H9c2 cells induced by DOX. The addition of Nrf2 signaling inhibitor ML385 reconfirmed that propofol may inhibit ferroptosis by activating Nrf2/GPx4 signaling.

Apoptosis, oxidative stress and inflammation are key aspects of DOX-induced cardiomyocyte injury [34]. In DOX-induced cardiomyocyte injury, Nrf2 induces upregulation of Hmox1, catalyzes heme degradation and promotes the release of Fe^2+^, which accumulates in mitochondria and triggers lipid peroxidation [7]. While the overproduction of ROS not only leads to apoptosis but also directly promotes the expression of inflammatory factors such as tumor necrosis factor-α (TNF-α) and interleukin-1β (IL-1β) [35,36]. Control of cardiac inflammation has been reported to be sufficient to attenuate cardiomyocyte death and cardiac dysfunction after DOX injection [16,37]. In this paper, we found that decreased cell viability and increased apoptotic cells, elevated level of oxidative stress, and increased levels of inflammatory factors after DOX treatment indicated that DOX treatment did trigger apoptosis, oxidative stress and inflammation in H9c2 cells. Interestingly, Nrf2 not only regulates mitochondrial function and thereby modulates the process of iron toxicity, but also protects cells from oxidative stress by modulating endogenous antioxidant response pathways [38]. Strong evidence also suggests that antioxidant therapy may reduce or prevent Dox-induced cardiotoxicity [39]. Accordingly, in addition to activating Nrf2 signaling, propofol has important antioxidant and anti-apoptotic effects. For example, propofol inhibits the expression of Bcl-2 and Bax [40]. Propofol protects against ROS-mediated cell damage by inhibiting ROS production [41]. Propofol reduces IL-1, IL-6 and TNF-α production in LPS-induced cardiomyocyte injury [42]. As shown in this study, in the DOX+Prop group, the decreased levels of apoptosis, oxidative stress marker ROS and inflammatory factors TNF-α, IL-6, IL-1β. What is more, ML385 reversed the inhibitory effects of propofol on H9c2 cells induced by DOX. Collectively, propofol inhibited the apoptosis, oxidative stress and inflammation in DOX-induced H9c2 cells by activating Nrf2/GPx4 signaling pathways. However, there are several limitations in this study. We explored the effects and the mechanism of propofol in H9c2 cells, but the influences of propofol on DOX-induced cardiomyocyte injury in animals and human is still to be investigated. In addition, besides the Nrf2/GPx4 signaling pathway, there may be other mechanisms of action by which propofol protects from DOX-induced cardiomyocyte injury, therefore more mechanisms of propofol need to be explored in further study.

## Conclusion

The current findings clearly support that appropriate concentration of propofol (20 μM) attenuates ferroptosis by activating Nrf2/GPx4 signaling pathways, thereby reducing DOX-induced cardiomyocyte injury. Given that several regulatory mechanisms and signaling pathways governing cell death such as Ripk3 have also been implicated in cardiac injury [43,44]. Therefore, research is also needed in the future to determine whether propofol regulates the Ripk3 signaling to affect DOX-induced cardiomyocyte injury.
